# Loneliness and Risk of Parkinson Disease

**DOI:** 10.1001/jamaneurol.2023.3382

**Published:** 2023-10-02

**Authors:** Antonio Terracciano, Martina Luchetti, Selin Karakose, Yannick Stephan, Angelina R. Sutin

**Affiliations:** 1 Department of Geriatrics, Florida State University College of Medicine, Tallahassee; 2 Department of Behavioral Sciences and Social Medicine, Florida State University College of Medicine, Tallahassee; 3 Euromov, Unité de Formation et de Recherche en Sciences et Techniques des Activités Physiques et Sportives, Université de Montpellier, Montpellier, France

## Abstract

**Importance:**

Loneliness is associated with morbidity and mortality, including higher risk of neurodegenerative diseases. To our knowledge, no study has examined whether the association between loneliness and detrimental outcomes extends to Parkinson disease (PD).

**Objective:**

To assess whether loneliness is associated with risk of incident PD and whether the association is independent of other risk factors or modified by age, sex, and genetic vulnerability.

**Design, Setting, and Participants:**

This prospective cohort study included a population-based sample of UK Biobank participants aged 38 to 73 years with loneliness data and without a diagnosis of PD at baseline who were first assessed from March 13, 2006, to October 1, 2010, and followed up to October 9, 2021.

**Exposure:**

Feeling lonely and covariates that are known risk factors for or prodromal features of PD.

**Main Outcome and Measure:**

Incident PD was ascertained through UK National Health Service health records.

**Results:**

Of 491 603 participants (mean [SD] age, 56.54 [8.09] years; 54.4% female), 2822 developed PD during the 15-year follow-up. Individuals who reported being lonely had a higher risk of PD (hazard ratio [HR], 1.37; 95% CI, 1.25-1.51), an association that remained after accounting for demographic factors, socioeconomic status, social isolation, PD polygenetic risk score, smoking, physical activity, body mass index, diabetes, hypertension, stroke, myocardial infarction, depression, and ever seeing a psychiatrist (fully adjusted model: HR 1.25; 95% CI, 1.12-1.39). The association between loneliness and incident PD was not moderated by sex (HR for interaction, 0.98; 95% CI, 95% CI, 0.81-1.18), age (HR for interaction, 0.99; 95% CI, 0.98-1.01), or polygenic risk score (HR for interaction, 0.93; 95% CI, 0.85-1.02). Contrary to expectations for a prodromal syndrome, when stratified by time, loneliness was not associated with risk for incident PD during the first 5 years (HR, 1.15; 95% CI, 0.91-1.45) but was associated with PD risk during the subsequent 10 years (HR, 1.32; 95% CI, 1.19-1.46).

**Conclusions and Relevance:**

This large cohort study found that loneliness was associated with risk of incident PD across demographic groups and independent of depression and other prominent risk factors and genetic risk. The findings add to the evidence that loneliness is a substantial psychosocial determinant of health.

## Key Points

**Question:**

Is loneliness associated with an increased risk of developing Parkinson disease?

**Findings:**

In this cohort study of 491 603 participants followed up for up to 15 years, loneliness was significantly associated with an increased risk of incident Parkinson disease independent of demographic and socioeconomic factors, social isolation, genetic risk, and physical and mental health.

**Meaning:**

The findings suggest that loneliness is associated with increased risk of developing Parkinson disease.

## Teaser

This cohort study uses data from the UK Biobank to assess the association of loneliness with the risk of incident Parkinson disease.

## Introduction

In May 2023, the surgeon general of the US released a report on loneliness, morbidity, and mortality^1^ that followed similar recommendations from the World Health Organization and the US National Academies of Sciences, Engineering, and Medicine.^2,3^ Loneliness, defined as a distressing subjective feeling that arises from the discrepancy between one’s desired and perceived social relationships, is characterized by heightened emotional vulnerability, hypervigilance, and perseverative cognition.^2,4^ In addition to its emotional toll, individuals who feel lonely tend to engage in unhealthy lifestyles and have worse clinical profiles.^1,2,3,5^ In part through emotional (eg, depression), behavioral (eg, physical inactivity), and biomedical (eg, diabetes) stressors, loneliness can harm brain health and is associated with increased risk of psychiatric and neurodegenerative diseases.^1,2,3,6^ Indeed, loneliness has been associated with neurological conditions, such as Alzheimer disease and related dementias.^7,8^ However, to our knowledge, there is no longitudinal evidence on whether individuals who report loneliness are at greater risk of developing Parkinson disease (PD). A fast-growing condition and the second most common neurodegenerative disease, PD is complex and includes heterogeneous presentations of motor and nonmotor symptoms.^9^ This study examined the prospective association between loneliness and incident PD over a 15-year follow-up period in a population-based UK Biobank cohort. The study further tested whether the association between loneliness and PD was moderated by age, sex, or genetic risk and whether the association was accounted for by other risk factors, including sociodemographic factors; indicators of behavioral (eg, physical activity), mental (eg, depression), physical (eg, diabetes), and social (eg, social isolation) health; or genetic risk.

## Methods

### Participants

The UK Biobank^10^ is an ongoing population-based cohort study. Individuals registered with the UK National Health Service (NHS) were recruited at 22 assessment centers across the UK between March 13, 2006, and October 1, 2010, when loneliness was assessed. Incident PD was ascertained through linked health records from NHS data available to October 9, 2021. Individuals were excluded if they withdrew from the study, had a PD diagnosis at baseline, or responded “prefer not to answer” or “do not know” or did not respond to the loneliness item. All UK Biobank participants gave written informed consent, which was approved by the North West Multicenter Research Ethics Committee. The current study was approved by the Florida State University Institutional Review Board with a waiver of consent due to secondary analyses of deidentified UK Biobank data. The study followed the Strengthening the Reporting of Observational Studies in Epidemiology (STROBE) reporting guideline.

### Measures and Outcome Ascertainment

#### Loneliness

Loneliness was measured with the item, “Do you often feel lonely?” Responses were coded as 0 for no and 1 for yes. Previous research^7^ has found that this item has a similar association with dementia risk as continuous multi-item scales.

#### PD

This study used the PD diagnosis and dates generated by the UK Biobank Outcome Adjudication Group (January 2022). As reported by the UK Biobank,^11^ incident PD was ascertained through linked NHS hospital admission and death records (using the earliest recorded date irrespective of source). The UK Biobank also used self-reports from nurse-led interviews to ask whether participants had been diagnosed with PD by a physician. All self-reported PD cases at baseline (prevalent PD) were excluded from the analyses of incident PD. Most incident cases were determined as the earliest known relevant *International Classification of Diseases*, *Ninth Revision* code 3320 or *International Statistical Classification of Diseases and Related Health Problems, Tenth Revision* code G20 in the hospital admission records. A small proportion of cases were ascertained from death register records. For those participants, the date of death was used as the date of ascertainment. In a validation study of 20 000 UK Biobank participants, it was found that the PD codes across all sources had a 91% positive predictive value.^11^

#### Covariates

All analyses included age and sex as covariates. The additional covariates were relevant to loneliness and PD, could be considered confounding factors or mediators, and have been used in relevant previous studies.^7,12,13,14,15,16^ Education was coded as a college or university degree or equivalent. The Townsend Deprivation Index^17^ was used as a proxy for socioeconomic status; scores were assigned to participants based on their postal codes, with higher scores indicating fewer resources. Never smokers were compared with current and former smokers based on questions about current (“Do you smoke tobacco now?”) and past (“In the past, how often have you smoked tobacco?”) tobacco smoking. Physical activity was measured with the mean score of 3 items from the International Physical Activity Questionnaire (eg, “In a typical week, on how many days did you walk for at least 10 minutes at a time?”).^18^

Body mass index (BMI) was calculated from standing height and weight measured at the assessment center. Diabetes was assessed with the question, “Has a doctor ever told you that you have diabetes?” The question, “Has a doctor ever told you that you have had any of the following conditions? (you can select more than one answer),” was used to assess hypertension, stroke, and heart attack (no [0]; yes [1]). Depression was assessed as a score of 3 or higher on the 2-item Patient Health Questionnaire.^19^ History of psychiatric services was assessed with the item, “Have you ever seen a psychiatrist for nerves, anxiety, tension, or depression?” (no [0]; yes [1]). Social isolation was the sum of 3 items: household size (lives with others [0]; lives alone [1]), frequency of visits with family or friends (at least once per month [0]; less than once per month [1]), and frequency of leisure and/or social activities (at least weekly participation [0]; no participation at least weekly in any activity [1]). Standard PD polygenic risk scores were obtained as described elsewhere.^20^

### Statistical Analysis

Descriptive statistics for study variables at baseline were computed as means and SDs or proportions for the full sample, by loneliness at baseline, and by incident PD. The association between loneliness and risk of incident PD was tested using Cox proportional hazards regression (reported using hazard ratios [HRs] and 95% CIs). Time was coded from the date of the loneliness assessment to the date of the first PD diagnosis, the date of death, or the censoring date (October 10, 2021). All tested models included age and sex as covariates. Model 1 included only age and sex; model 2 included college education and the Townsend Deprivation Index; model 3 included social isolation; model 4 included the PD polygenic risk score; model 5 included current smoking, former smoking, and physical activity; model 6 included BMI, diabetes, hypertension, stroke, and myocardial infarction (MI); model 7 included depression and history of psychiatric services; and model 8 was fully adjusted by including all covariates. To gauge to what extent the covariates in each model explained the association between loneliness and risk of incident PD, we used the following formula for percentage of excess risk mediated^13^: [(HR_model 1_ – HR_model >1_) / (HR_model 1_ – 1)] × 100.

Because the incidence of PD varies by sex, age, and genetic risk, additional analyses examined whether these variables moderated the association between loneliness and PD by testing interaction terms in model 1 (eg, sex × loneliness). Sensitivity analyses further examined the robustness of the findings. First, because PD is more common in older adults, we excluded participants younger than 50 years at the baseline assessment. Second, to better identify the temporal relationship between loneliness and PD, the analyses were repeated stratifying time from baseline to 5 years and from 5 to 15 years.

The statistical analyses were performed between April and June 2023 using SPSS, version 27 (IBM Corp) and Stata, version 16 (StataCorp LLC). Two-sided *P* < .05 was considered statistically significant.

## Results

Of 502 505 individuals recruited, 10 902 were excluded: 138 withdrew from the study, 896 had a PD diagnosis at baseline, 1429 responded “prefer not to answer” and 7531 “do not know” to the loneliness item, and 908 did not respond to the loneliness item. A total of 491 603 individuals were included in the study. At the baseline assessment, participants ranged in age from 38 to 73 years (mean [SD] age, 56.54 [8.09] years); 54.4% were female, and 45.6% were male (Table). Compared with individuals who did not report being lonely (400 417 [81.5%]), individuals who reported being lonely (91 186 [18.5%]) were slightly younger, were more likely to be female, and had fewer resources (more social isolation, higher deprivation, and less likely to have a college degree), more health risk behaviors (more likely to be a current smoker and physically inactive), worse physical health (more likely to have diabetes, hypertension, MI, and stroke), and worse mental health (more likely to have depression or to have seen a psychiatrist for anxiety or depression).

**Table.** Descriptive Statistics for the Full Sample and by Baseline Loneliness and Incident PD

Characteristic	Participants^a^				
	Total (N = 491 603)	Loneliness at baseline		Incident PD	
		No (n = 400 417)	Yes (n = 91 186)	No (n = 488 781)	Yes (n = 2822)
Follow-up, mean (SD), y	12.33 (1.80)	12.35 (1.78)	12.26 (1.92)	12.35 (1.77)	8.61 (2.99)
Reported loneliness	91 186 (18.5)	0	91 186 (100)	90 637 (18.5)	549 (19.5)
Sex					
Female	267 529 (54.4)	210 580 (52.6)	56 949 (62.5)	266 476 (54.5)	1053 (37.3)
Male	224 074 (45.6)	189 837 (47.4)	34 237 (37.5)	222 305 (45.5)	1769 (62.7)
Age, mean (SD), y	56.54 (8.09)	56.77 (8.08)	55.54 (8.05)	56.50 (8.09)	63.15 (5.18)
College education	158 498 (32.2)	133 830 (33.4)	24 668 (27.1)	157 733 (32.3)	765 (27.1)
Townsend Deprivation Index score, mean (SD)	−1.32 (3.08)	−1.50 (2.98)	−0.52 (3.39)	−1.32 (3.08)	−1.38 (3.06)
Smoking status					
Never	268 006 (54.5)	221 340 (55.3)	46 666 (51.2)	266 558 (54.5)	1448 (51.3)
Former	170 113 (34.6)	140 076 (35.0)	30 037 (32.9)	168 948 (34.6)	1165 (41.3)
Current	51 697 (10.5)	37 639 (9.4)	14 058 (15.4)	51 503 (10.5)	194 (6.9)
Physical activity score, mean (SD)	3.65 (1.64)	3.68 (1.62)	3.55 (1.72)	3.65 (1.64)	3.67 (1.64)
BMI, mean (SD)	27.43 (4.80)	27.29 (4.64)	28.06 (5.40)	27.43 (4.80)	27.79 (4.58)
Diabetes	25 733 (5.2)	19 371 (4.8)	6362 (7.0)	25 419 (5.2)	314 (11.1)
Hypertension	133 050 (27.1)	106 179 (26.5)	26 871 (29.5)	131 949 (27.0)	1101 (39.0)
Myocardial infarction	11 342 (2.3)	8821 (2.2)	2521 (2.8)	11 220 (2.3)	122 (4.3)
Stroke	7459 (1.5)	5525 (1.4)	1934 (2.1)	7356 (1.5)	103 (3.6)
PD polygenic risk score, mean (SD)	−0.14 (1.02)	−0.14 (1.02)	−0.15 (1.02)	−0.14 (1.02)	0.20 (1.03)
Social isolation score, mean (SD)	0.57 (0.67)	0.52 (0.64)	0.81 (0.75)	0.57 (0.67)	0.59 (0.71)
Depressed mood	27 125 (5.5)	10 998 (2.7)	16 127 (17.7)	26 968 (5.5)	157 (5.6)
Have seen a psychiatrist for anxiety or depression	56 092 (11.4)	34 841 (8.7)	21 251 (23.3)	55 639 (11.4)	453 (16.1)

Abbreviations: BMI, body mass index (calculated as weight in kilograms divided by height in meters squared); PD, Parkinson disease.

^a^ Data are presented as number (percentage) of participants unless otherwise indicated.

Over the course of the study, which spanned 15.58 years (mean [SD], 12.33 [1.80] years; 6 062 197 person-years), 2822 participants developed PD (incidence rate, 2822 per 6 062 197 person-years; 47 per 100 000 person-years), including 2273 (incidence rate, 46 per 100 000 person-years) in the group that did not report loneliness and 549 (incidence rate, 49 per 100 000 person-years) in the group that reported loneliness. The 2822 individuals who were diagnosed with PD were older; more likely to be male; to be former smokers; to have a higher BMI; to have a higher PD polygenetic risk score; to have a diagnosis of diabetes, hypertension, MI, or stroke; and to have seen a psychiatrist for anxiety or depression compared with individuals who were not diagnosed with PD by the censoring date (n = 488 781). They were less likely to have a college degree or to be current smokers (Table).

**Figure 1.**
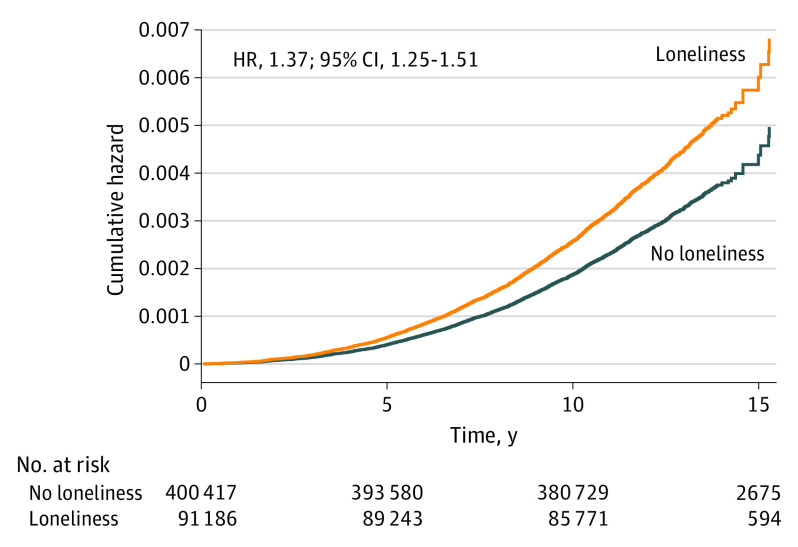
Cumulative Hazard of Parkinson Disease Among Participants Who Did and Did Not Report Loneliness at Baseline, Adjusted for Age and Sex.

**Figure 2.**
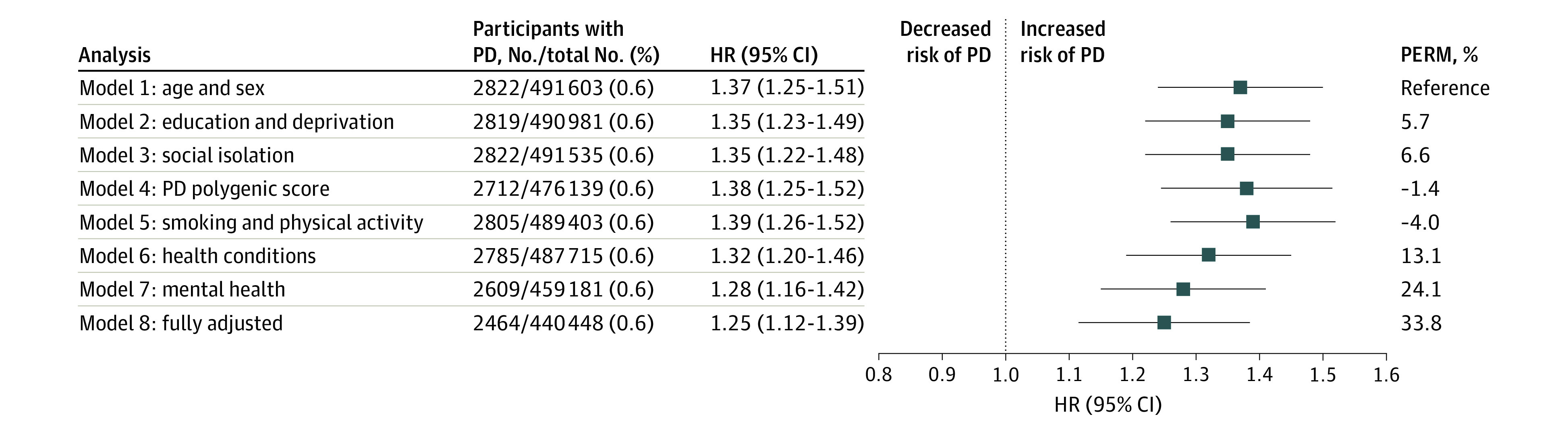
Association of Loneliness With Incident Parkinson Disease (PD) in Models With Different Sets of Covariates and the Fully Adjusted Model Numbers of total participants and participants with PD vary across models because of missing data for the covariates. The percentage of excess risk mediated (PERM) represents the level of attenuation of other models compared with the basic model (model 1). Age and sex were included as covariates in all models. Markers indicate hazard ratios (HRs), with horizontal lines representing 95% CIs.

The primary analysis indicated that individuals who reported being lonely had a higher risk of PD (HR, 1.37; 95% CI, 1.25-1.51) (Figure 1), an association that remained across all models (Figure 2 and eTable in Supplement 1). The comparisons of model 1 (age and sex) with model 2 (education and deprivation index), 3 (social isolation), 4 (PD polygenic risk score), and 5 (smoking and physical activity) indicated that most covariates did not substantially attenuate the association between loneliness and PD risk. However, the association between loneliness and PD was attenuated by 13.1% with the health variables (BMI, diabetes, hypertension, MI, and stroke; model 6: HR 1.32; 95% CI, 1.20-1.46), by 24.1% with the mental health variables (depression, ever seen a psychiatrist; model 7: HR, 1.28; 95% CI, 1.16-1.42), and by 33.8% in the fully adjusted model (model 8: HR, 1.25; 1.12-1.39). However, in the fully adjusted model, the association between loneliness and PD remained. Notably, in model 3, social isolation was not associated with PD risk (HR, 1.05; 95% CI, 0.99-1.11).

In follow-up analyses, there was no significant interaction between loneliness and sex (overall: HR for interaction, 0.98 [95% CI, 0.81-1.18]; female: HR, 1.39 [95% CI, 1.21-1.59]; male: HR, 1.36 [95% CI, 1.20-1.55]), loneliness and age (HR, 0.99; 95% CI, 0.98-1.01), or loneliness and PD polygenic risk score (HR for interaction, 0.93; 95% CI, 0.85-1.02). Sensitivity analysis excluding individuals younger than 50 years confirmed the association (2767 of 376 606 [0.7%] had PD; HR, 1.36; 95% CI, 1.24-1.50). In analyses stratified by follow-up time, loneliness was not associated with incident PD during the first 5 years after baseline (388 of 8780 [4.4%] had PD; HR, 1.15; 95% CI, 0.91-1.45), but it was during the subsequent 5 to 15 years of follow-up (2341 of 481 499 [0.5%] had PD; HR, 1.32; 95% CI, 1.19-1.46).

## Discussion

In this population-based cohort study, participants who reported loneliness were at higher risk of developing PD during the 15-year follow-up. Loneliness was associated with a higher risk of subsequent PD after accounting for basic demographic variables and other potential risk, protective, prodromal, or confounding factors, including social isolation, socioeconomic status, genetic risk, smoking, physical activity, diabetes and hypertension, depression, and having seen a psychiatrist for anxiety or depression symptoms. While incidence of PD varies by age, sex, and genetic risk, the association between loneliness and PD was similar in males and females and across age and polygenic risk scores. The association was specific to the subjective experience of loneliness and was not observed for the objective measure of social isolation.

To our knowledge, this is the first study to examine the association between loneliness and risk of subsequent PD. Our findings complement other evidence that loneliness is a psychosocial determinant of health^21^ associated with increased risk of morbidity and mortality. For example, a meta-analysis^8^ found that loneliness was associated with a 23% increased risk of dementia. Expanding on the meta-analysis,^8^ a UK Biobank study^7^ that used the same loneliness measure as the present study found that feeling lonely was associated with an increased risk of Alzheimer disease and vascular and frontotemporal dementia. The current and previous findings suggest that loneliness may be associated with increased risk of neurodegenerative diseases and that the detrimental effects of loneliness are not limited to a single etiologic or neuropathologic pathway. Indeed, we are not aware of evidence that links loneliness to proteins like α-synuclein or brain structures that play a major role in PD, such as basal ganglia and substantia nigra.^22^

### Mechanisms

There are several plausible and non–mutually exclusive interpretations of the observed association between loneliness and PD. For example, the association could be spurious and due to unaccounted for confounding factors or residual confounding due to imprecisely measured covariates. The association could be due to shared risk factors like genetic factors or mental health conditions. The finding that the PD polygenic risk score did not attenuate the association suggests that shared genetic factors are unlikely to play a substantial role in the observed association. Similarly, the findings indicated that while chronic conditions and especially mental health accounted for part of the association, loneliness still had an independent association with PD risk.

Another possible interpretation is that PD neuropathology may be associated with a rise in loneliness in the preclinical or prodromal stages of the disease (ie, reverse causality). Indeed, nonmotor symptoms (eg, depression, fatigue, anxiety, or apathy) are common in PD and can emerge early in the disease process. However, while greater loneliness is a concern for people with PD,^23,24,25^ 1 cross-sectional study^26^ found no differences in loneliness between individuals with or without PD. Furthermore, our results suggest that this reverse causality interpretation is unlikely to fully explain the association. The association, for example, remained after accounting for depression, which suggests that it was not due to the shared overlap with this prodromal symptom. In addition, contrary to what would be expected by reverse causality, loneliness was not associated with incident PD within the first 5 years, but it was associated with incident PD in the subsequent 10 years.

The most probable (and parsimonious) interpretation is that loneliness is a risk factor for PD through various pathways. This study tested a broad range of covariates that could be potential mediators. Individuals who experience loneliness tend to engage in detrimental behaviors, such as physical inactivity, but this pathway seems unlikely to play a major role given that the association between loneliness and PD was unchanged with the inclusion of 2 prominent health behaviors. Loneliness seems more likely to be associated with increased risk of PD through metabolic, inflammatory, and neuroendocrine pathways,^27,28^ as the association was attenuated by 13.1% after accounting for chronic conditions, such as diabetes. Not surprisingly, the association of loneliness with PD was attenuated the most (by 24.1%) with the inclusion of mental health variables in the model. Longitudinal evidence suggests that there are bidirectional associations between loneliness and depression,^29,30^ which are likely to co-occur and share pathways with increased risk of PD. Still, our findings indicated that loneliness remained associated with PD after accounting for mental health variables.

There are likely to be other pathways that contribute to the risk of PD associated with loneliness, such as microglia-mediated neuroinflammation.^5,27,31^ It would be fruitful to examine whether loneliness is associated with markers of neuropathology, such as α-synuclein and neurofilament light chain. Of note, loneliness could be directly associated with the risk of neuropathology but could also be associated with increased risk of PD by eroding resilience against neurodegenerative processes that contribute to the development of PD.

### Future Directions

In addition to better identifying mechanisms that explain why loneliness is associated with increased risk of PD, there are other worthwhile directions for research on loneliness and PD. For example, this research focused on loneliness as a risk factor for incident PD, but longitudinal research could help determine whether there are changes in loneliness before and after the onset of PD, which has implications for understanding loneliness as a risk factor as well as a potential sequela of PD. Furthermore, given the observed association and the implications of loneliness for a broad range of health outcomes, effective psychosocial interventions to reduce loneliness are needed.^32^ In addition to the potential for primary prevention, among patients with PD, addressing loneliness may be associated with reduced risk of dementia^7^ and improved quality of life.^23,24,33^

### Strengths and Limitations

The major strengths of the study include the large sample size and statistical power, the long follow-up, the broad range of covariates to account for relevant risk factors, and the independent ascertainment of diagnoses based on health records. There are also limitations. This observational study could not determine causality or whether reverse causality could explain the observed associations. Loneliness was assessed by a single yes or no item. This measure is reliable and valid,^7,14^ but compared with multi-item scales, the single-item assessment is likely to increase error variance and underestimate the association between loneliness and PD. Another limitation is the use of hospital admission and death records, which are likely to miss PD at the early stages. It is possible that some participants were diagnosed with PD during follow-up but were not hospitalized; these participants would be incorrectly censored as not having PD in our analyses. This inaccurate classification could potentially underestimate the association between loneliness and risk of PD. It is essential to test whether these findings can be replicated using different ascertainment methods. However, the ascertainment via health records is independent from the research study participation, avoiding the attrition biases typical of longitudinal studies. The sample was relatively young, but there was no age interaction with loneliness, and excluding younger participants had no effect on the estimated effect size. Also, the UK Biobank is not a representative sample, and the response rate was 5.5%^34^; however, risk factor associations in the UK Biobank are similar to those found in representative samples.^34^ While the UK health care system provides several advantages (eg, relatively uniform access to high-quality health care), it remains to be addressed whether the findings generalize to other contexts, including middle- and low-income countries.

## Conclusions

This cohort study found that individuals who felt lonely had greater risk of PD regardless of genetic and established behavioral, social, and clinical risk factors. This study adds evidence on the detrimental health impact of loneliness and supports recent calls^1^ for the protective and healing effects of personally meaningful social connection.

## Notes

Supplement 1. **eTable.** Hazard Ratios of the Association of Loneliness With Incident Parkinson Disease (PD) Accounting for Different Sets of Covariates and Fully Adjusted Model

Supplement 2.
Data Sharing Statement
