# Supplementary material for: Loneliness and Risk of Parkinson Disease
Source: JAMA Neurol. 2023 Oct 2;80(11):1138–44. doi: 10.1001/jamaneurol.2023.3382 (PMC10546293; doi:10.1001/jamaneurol.2023.3382)
Supplement: Supplement 1. — eTable. Hazard Ratios of the Association of Loneliness With Incident Parkinson Disease (PD) Accounting for Different Sets of Covariates and Fully Adjusted Model [file jamaneurol-e233382-s001.pdf]

Supplemental Online Content

Terracciano A, Luchetti M, Karakose S, Stephan Y, Sutin AR. Loneliness and risk of Parkinson disease. *JAMA Neurol*. Published online October 2, 2023. doi:10.1001/jamaneurol.2023.3382

eTable. Hazard Ratios of the Association of Loneliness With Incident Parkinson Disease (PD) Accounting for Different Sets of Covariates and Fully Adjusted Model

This supplementary material has been provided by the authors to give readers additional information about their work

© 2023 Terracciano A et al. *JAMA Neurology.*

eTable. Hazard Ratios of the Association of Loneliness With Incident Parkinson Disease (PD) Accounting for Different Sets of Covariates and Fully Adjusted Model

	Model 1			Model 2			Model 3			Model 4			Model 5			Model 6			Model 7			Model 8		
	HR	L	H	HR	L	H	HR	L	H	HR	L	H	HR	L	H	HR	L	H	HR	L	H	HR	L	H
**Loneliness**	1.37	1.25	1.51	1.35	1.23	1.49	1.35	1.22	1.48	1.38	1.25	1.52	1.39	1.26	1.52	1.32	1.20	1.46	1.28	1.16	1.42	1.25	1.12	1.39
**Sex**	2.01	1.86	2.17	2.01	1.86	2.17	2.01	1.86	2.17	2.04	1.89	2.21	2.04	1.89	2.21	1.95	1.80	2.10	2.01	1.86	2.18	2.03	1.86	2.21
**Age**	1.16	1.15	1.16	1.16	1.15	1.16	1.16	1.15	1.16	1.16	1.15	1.16	1.16	1.15	1.16	1.15	1.15	1.16	1.16	1.15	1.17	1.16	1.15	1.17
**Townsend deprivation**				1.02	1.00	1.03																1.01	0.99	1.02
**College**				0.99	0.91	1.07																0.96	0.88	1.05
**Social isolation**							1.05	0.99	1.11													1.05	0.99	1.12
**PGS_PD**										1.39	1.34	1.45										1.40	1.35	1.46
**Smoking, current**													0.76	0.66	0.89							0.68	0.58	0.81
**Smoking, former**													0.94	0.87	1.01							0.92	0.84	1.00
**Physical activity**													0.97	0.94	0.99							0.97	0.95	0.99
**Diabetes**																1.55	1.36	1.75				1.50	1.31	1.72
**Stroke**																1.54	1.26	1.88				1.51	1.21	1.87
**Heart attack**																0.95	0.79	1.15				0.96	0.79	1.17
**Hypertension**																1.08	1.00	1.17				1.04	0.96	1.14
**Body mass index (BMI)**																1.00	0.99	1.01				1.00	0.99	1.01
**Depressed**																			1.21	1.02	1.44	1.14	0.95	1.37
**Seen a psychiatrist for nerves, anxiety, tension or depression**																			1.52	1.37	1.70	1.47	1.31	1.65

Notes: L (lower) and U (upper) refer to the 95% confidence interval. PGS = Polygenic score.

© 2023 Terracciano A et al. *JAMA Neurology.*
