# Supplementary material for: Loneliness and Risk of Parkinson Disease
Source: JAMA Neurol. 2023 Oct 2;80(11):1138–44. doi: 10.1001/jamaneurol.2023.3382 (PMC10546293; doi:10.1001/jamaneurol.2023.3382)
Supplement: Supplement 2. — Data Sharing Statement [file jamaneurol-e233382-s002.pdf]

Data Sharing Statement

Terracciano. Loneliness and Risk of Parkinson Disease. *JAMA Neurol*. Published October 02, 2023. doi:10.1001/jamaneurol.2023.3382

Data
Data available: Yes
Data types: Deidentified participant data, Data dictionary
How to access data: The UK Biobank data are available to researchers who apply with UK Biobank (https://www.ukbiobank.ac.uk). Key elements of the application are a research plan, a listing of requested variables, and a payment to the UK Biobank.
When available: With publication

Supporting Documents
Document types: None

Additional Information
Who can access the data: UK Biobank data are available to researchers who apply with UK Biobank (https://www.ukbiobank.ac.uk).
Types of analyses: UK Biobank data are available to researchers who apply with UK Biobank (https://www.ukbiobank.ac.uk).
Mechanisms of data availability: after approval of a proposal by UK Biobank
